# Antifungal Activity and Molecular Mechanisms of Partial Purified Antifungal Proteins from *Rhinacanthus nasutus* against *Talaromyces marneffei*

**DOI:** 10.3390/jof6040333

**Published:** 2020-12-03

**Authors:** Juthatip Jeenkeawpieam, Supachai Yodkeeree, Alex Andrianopoulos, Sittiruk Roytrakul, Monsicha Pongpom

**Affiliations:** 1 Department of Microbiology, Faculty of Medicine, Chiang Mai University, Chiang Mai 50200, Thailand; juthatip_jee@cmu.ac.th; 2 Department of Biochemistry, Faculty of Medicine, Chiang Mai University, Chiang Mai 50200, Thailand; supachai.y@cmu.ac.th; 3 Molecular, Cellular and Developmental Biology, School of Biosciences, University of Melbourne, 3010 Victoria, Australia; alex.a@unimelb.edu.au; 4 Functional Ingredients and Food Innovation Research Group, National Center for Genetic Engineering and Biotechnology, Pathum Thani 12120, Thailand; sittiruk@biotec.or.th

**Keywords:** antifungal protein, AFP, *Talaromyces marneffei*, *Rhinacanthus nasutus*, G-protein

## Abstract

Antifungal proteins (AFPs) are able to inhibit a wide spectrum of fungi without significant toxicity to the hosts. This study examined the antifungal activity of AFPs isolated from a Thai medicinal plant, *Rhinacanthus nasutus,* against the human pathogenic fungus *Talaromyces* *marneffei*. This dimorphic fungus causes systemic infections in immunocompromised individuals and is endemic in Southeast Asian countries. The *R. nasutus* crude protein extract inhibited the growth of *T. marneffei*. The anti-*T. marneffei* activity was completely lost when treated with proteinase K and pepsin, indicating that the antifungal activity was dependent on a protein component. The total protein extract from *R. nasutus* was partially purified by size fractionation to ≤10, 10–30, and ≥30 kDa fractions and tested for the minimal inhibitory concentration (MIC) and minimal fungicidal concentration (MFC). All fractions showed anti-*T. marneffei* activity with the MIC and MFC values of 32 to 128 μg/mL and >128 μg/mL, respectively. In order to determine the mechanism of inhibition, all fractions were tested with *T. marneffei* mutant strains affected in G-protein signaling and cell wall integrity pathways. The anti-*T. marneffei* activity of the 10–30 kDa fraction was abrogated by deletion of *gasA* and *gasC*, the genes encoding alpha subunits of heterotrimeric G-proteins, indicating that the inhibitory effect is related to intracellular signaling through G-proteins. The work demonstrates that antifungal proteins isolated from *R. nasutus* represent sources for novel drug development.

## 1. Introduction

*Talaromyces marneffei* causes a life-threatening mycosis (talaromycosis), primarily affecting immunocompromised residents and travellers in Southeast Asia, southern China, and northeastern India [1,2]. Amphotericin B and itraconazole are effective drugs in the treatment of talaromycosis [3,4,5]. Other azoles such as voriconazole are also effective, whereas echinocandins such as anidulafungin showed only moderate activities against the pathogenic yeast phase of *T. marneffei* [6,7,8]. In recent years, resistance to some antifungal drugs has been reported for *T. marneffei*, especially for fluconazole [3,4,5,6,7,8]. Moreover, an increasing number of *T. marneffei* infections have been reported among non-HIV-infected patients with other immunocompromising conditions [9]. Therefore, the discovery of new antifungal drugs is necessary to cover the shifting challenges of this and other fungal infections.

Natural selection has led to the evolution of antimicrobial peptides (AMP) and these act as the first line of defences in hosts to surrounding pathogens [10,11]. This study focused on one of the AMPs, antifungal peptides (AFPs). AFPs have been reported from many sources, including insects, plants, and mammals [12,13,14,15]. They are small, cationic, and amphipathic molecules, and many were shown to be neither cytotoxic nor immunogenic on different types of mammalian cells, hence indicating their potential for medical applications [12,13,14,15]. Thus, these AFPs serve as a major source of new drugs.

The shrub *Rhinacanthus nasutus* (L.) Kurz. has been widely used for treatment of several diseases in Thai traditional medicine including cancers, eczema, pulmonary tuberculosis, herpes, hepatitis, diabetes, hypertension, and skin diseases [16]. Several active groups of compounds have been identified in *R. nasutus* extracts such as steroids, flavonoids and naphthoquinone. Among these, the naphthoquinone rhinacanthins was found to have major anti-cancer properties [17]. *R. nasutus* extracts have also been demonstrated to contain antifungal activity [18]. These extracts showed inhibition of several human pathogenic fungi, including *Microsporum gypeseum*, *M. canis*, *Trichophyton rubrum*, *T. mentagrophytes*, *Epidermophyton floccosum*, *Candida albicans*, *Cryptococcus neoformans*, *Aspergillus niger, Malassezia* sp., and *Magnaporthe oryzae* [19,20,21,22]. Rhinacanthins are believed to induce cell wall degeneration and cell lysis. However, the exact mechanism of antifungal activity is unknown [23]. Rhinacanthins themselves are organic compounds and they need to be solubilized in organic solvent, and as such are highly toxic and require special preparation for clinical application. Both ethanol and water-based extracts of *R. nasutus* also exhibit antifungal activity, with ethanol-based extracts showing greater activity [17]. This data suggests that there are protein-based compounds that contain the antifungal activity [21]. Currently, neither active protein compounds nor AFPs isolated from *R. nasutus* has been reported. We have previously demonstrated that a protein extract from *R. nasutus* possesses antifungal activity against *T. marneffei*, *C. neoformans,* and *A. fumigatus* [24]. In this study, we aimed to purify and characterize the AFPs in *R. nasutus* and examine the antifungal activity, including the molecular mechanism of action against the dimorphic opportunistic fungus *T. marneffei*. The outcome from this study may lead to the discovery of a novel AFP that can be used in therapeutic application for the treatment of opportunistic fungal infection.

## 2. Materials and Methods

### 2.1. Preparation of Protein Extract

Thirty grams of *R. nasutus* leaves were collected from the medicinal plant garden unit, Faculty of Pharmacy, Chiang Mai University, Thailand. They were cut, frozen in liquid nitrogen, ground to fine powder and solubilized in extraction buffer (10 mM sodium acetate buffer pH 5.2, 0.5% polyvinylpolypyrrolidone (PVPP)). The cell lysates were precipitated at 80% saturation level of ammonium sulphate. The precipitated protein was dialyzed in 10 mM sodium acetate buffer pH 5.2 at 4°C and the protein concentration was determined by Bradford assay (Bio-Rad Protein Assay; dye reagent concentrate, Bio-Rad, Hercules, CA, USA) [25].

### 2.2. Antifungal Activity Testing

The antifungal assay using a modified agar well diffusion method was performed as previously described [8,26,27]. The yeast pathogenic phase of *T. marneffei* was chosen for the testing. Briefly, a yeast colony of *T. marneffei* ATCC18224 was grown on synthetic dextrose (SD) agar (0.17% (*w*/*v*)) yeast nitrogen base without ammonium sulfate and amino acids, 2% (*w*/*v*) glucose, 10 mM (NH_4_)_2_SO_4_, 2% agar) at 37 °C for 7–10 days. The yeast cells were collected by scraping the colonies off the plate, resuspended in 1xPBS buffer, enumerated and adjusted to 10^8^ cells/mL RPMI-based agar was inoculated by spreading 100 µL of fungal suspension over the surface. Wells were created by puncturing the agar with a sterile borer (5 mm diameter). The well was filled with a 100 µL solution containing 200 µg of the protein extract. The plates were incubated at 37 °C for 72 h. This qualitative test included a positive control (10 µg/mL amphotericin B) and a negative control (10 mM sodium acetate buffer, pH 5.2). The antifungal effects were recorded as follows: −, no inhibition; +, inhibition, with recorded inhibition zones. The experiments were performed in triplicate.

### 2.3. Phenolic Compound Content and Proteolytic Digestion Assay

Measurement of phenolic compound content and proteolytic digestion assay was performed in order to confirm that the antifungal effect was derived from protein components. Total phenolic content was determined with the modified Folin-Ciocalteu method in a 96-well microplate using gallic acid as a standard. Briefly, the 40 µL sample was mixed with 60 µL of 10% Folin-Ciocalteu reagent. After incubating at room temperature for 5 min in the dark, 60 µL of 7.5% (*w*/*v*) Na_2_CO_3_ was added to each well and incubated at room temperature for 30 min. The absorbance of the reaction mixture was measured at 765 nm in a microplate reader (BioTek™ Synergy™ H4 Hybrid Microplate Reader, Winooski, VT, USA) [28]. Proteolytic digestion is performed by using 200 µg/mL proteinase K or porcine pepsin (enzyme to protein ratio, 1:25) at 37 °C for 12 h, followed by heat inactivation by boiling for 10 min [29]. The digested proteins were then used for antifungal activity testing.

### 2.4. Fractionation of The Antifungal Peptides

The protein extract was partial purified by size fractionation. The ammonium sulphate precipitated protein was initially centrifuged in a column with molecular weight cut-off of 30- kDa (GE Healthcare Life Sciences, NJ, USA). The upper fraction which contained proteins with molecular weight ≥30 kDa was collected (RN_A). Then the flow-through was loaded and centrifuged in a column with molecular weight cut-off of 10- kDa (GE Healthcare Life Sciences, NJ, USA). The upper fraction with molecular weight 10–30 kDa (RN_B) and the flow-through containing proteins ≤10 kDa (RN_C) were collected. Each fraction was then used for antifungal activity testing.

### 2.5. Anti-T. marneffei Activity Testing

A modified broth microdilution technique was used for this assay [8,30,31]. Briefly, 50 μL of each fraction containing 400 μg/mL protein was placed into a sterile 96-well microtiter plates. An equal volume of *T. marneffei* yeast cell suspension in RPMI 1640 medium (0.5–2.5 × 10^4^ cells/mL) was then added to each well. Plates were incubated at 37 °C for 72 h. Then a 0.18% (*w*/*v*, final concentration) resazurin (Sigma-Aldrich, MO, USA) indicator was added to each well [32]. Fungal growth was determined by measuring absorbance at 600 nm (BioTek™ Synergy™ H4 Hybrid Microplate Reader, Winooski, Vermont, USA). The controls included a growth control (fungus only), a medium control (medium only), and fungus with medium plus amphotericin B (10 µg/mL), which is used as a positive control for inhibition. The experiments were performed in triplicate.

### 2.6. Determination of The Minimal Inhibitory Concentration (MIC) and Minimal Fungicidal Concentration (MFC)

The MICs of the partial purified fractions were determined by a broth microdilution assay according to a modification of M27-A4 and M38-A3 protocols [8,30,31]. The tests were performed as previously mentioned in Section 2.5, except 2-fold serial dilutions of the crude extracts were prepared, yielding a protein concentration range of 0.25–128 μg/mL. Plates were incubated at 37 °C for 72 h. The MICs value were read at the lowest concentration of the extract that could inhibit the growth of *T. marneffei*. The results were interpreted based on a resazurin color change and the absorbance values at 600 nm. If there was no inhibition at the maximum concentration tested (128 μg/mL), an MIC of >128 μg/mL was recorded.

After the MIC values were read, the MFCs were determined by dropping a 10 μL aliquot from the wells corresponding to the MIC and higher protein concentrations onto a brain heart infusion agar (BHI) plate and incubating at 37 °C for 72 h. The protein concentration of the extract that showed no colonial growth of *T. marneffei* on BHI plates was recorded as the minimal fungicidal concentration (MFC). The value of >128 μg/mL was recorded if colonies were observed at the maximum concentration (128 μg/mL) tested.

### 2.7. Characterization of The Molecular Mechanism of Anti-T. marneffei Antifungal Proteins

*T. marneffei* strains used in this study are listed in Table 1. The *gasA*, *gasB* and *gasC* genes encode the three alpha subunits of heterotrimeric G-protein complexes, and the Δ*gasA*, Δ*gasB,* and Δ*gasC* strains are gene deletion mutants in each of these genes. The *slnA* and *drkA* genes encode hybrid histidine kinases and the Δ*slnA* and Δ*drkA* strains are gene deletion mutants in these genes. All strains were used in the broth microdilution antifungal activity testing assay.

The effect of *R. nasutus* fractions on strains mutated in the heterotrimeric G-protein signaling and SlnA/DrkA associated cell wall integrity pathways was investigated. Yeast cells from the wild type, Δ*gasA*, Δ*gasB*, Δ*gasC,* Δ*slnA*, and Δ*drkA* strains were prepared. They were seeded into the wells of a microtiter plate at 10^4^ conidia/mL and treated with each of RN_A, RN_B, and RN_C fractions at 4xMIC. This high amount of protein is used to ensure the inhibitory effect. The plates were incubated at 37 °C for 72 h. Then the resazurin indicator was added, and the OD600 nm was determined to assess growth.

To confirm the effect of the *R. nasutus* extract on the heterotrimeric G-protein signaling, two guanidine nucleotide analogues, guanosine-5′-(3-thiotriphosphate) (GTPγS) and guanosine-5′-*O*-(2-thio-bisphosphate) (GDPβS) were included in the antifungal assay [37]. The yeast cells of wild type, Δ*gasA*, Δ*gasB*, and Δ*gasC* strains were pretreated with 100 µM of the nucleotide analogs GTPγS, GDPβS and a control adenosine 5′-[γ-thio]triphosphate (ATPγS) (Sigma-Aldrich, Mannheim, Germany) at 37 °C for 15 min. Then a 4xMIC concentration of RN_B (256 µg/mL) was added. The plate was incubated at 37 °C for 72 h before addition of resazurin indicator and the OD600 nm was determined. The controls included a growth control (fungus only), a nucleotide analog control (fungus + each nucleotide analog) and fungus with medium plus amphotericin B (10 µg/mL) is used as a positive control for inhibition. The percentage of growth in the mutant strains was compared to the wild type. Experiments were performed in duplicate.

### 2.8. Statistical Analysis

All data were subjected to one-way analysis of variance, followed by Duncan’s multiple range test to find the level of significance in differences between the mean values. The normal distribution and homogeneity of variance of the data were checked by Shapiro–Wilk test and Levene’s test, respectively. Variability in the data was expressed as the standard error of the mean (SEM) and differences with *p* < 0.05 were considered significant. The data were analyzed using SPSS version 16.

## 3. Results

### 3.1. Protein Extract of R. nasutus Exhibited Antifungal Activity Against T. marneffei

**Figure 1:**
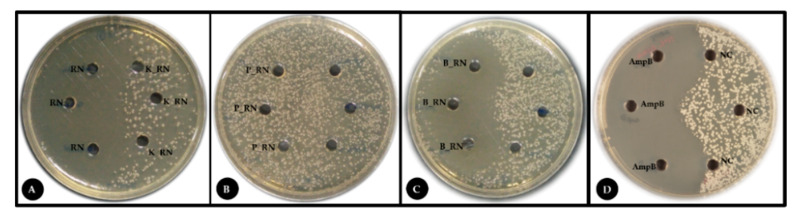
Antifungal activity of a protein extract from *R. nasutus*. (**A**) The ammonium sulphate precipitated *R. nasutus* protein extract (RN), proteinase digested samples (proteinase K (K_RN) and (**B**) pepsin (P_RN)), and (**C**) heat-inactivated precipitated protein (B_RN) were evaluated by agar well diffusion method against *T. marneffei*. The yeast cells were spread onto the surface of the agar medium and the tested reagent used to fill the appropriate wells. (**D**) Positive (10 µg/mL amphotericin B; AmpB) and negative (10 mM CH_3_COONa; NC) controls are shown. Plates were incubated at 37 °C for 72 h.

*R. nasutus* leaves were used to prepare a crude protein extract. A 100 µL aliquot containing 200 µg of the plant protein extract was used in an agar well diffusion assay against *T. marneffei* at 37 °C. At 37 °C, *T. marneffei* grows in a unicellular yeast form that represents the pathogenic form in human infections. Compared to the negative control where no inhibition of growth was evident, the *R. nasutus* extract showed inhibition similar to the amphotericin B positive control (Table 2). Phenolic compound content analysis of the crude and ammonium sulphate precipitated protein extract showed that they contained equivalent amounts of phenolic compounds as protein, 0.948 and 0.739 mg/gram, respectively. Testing of the proteinase digested extract, using either proteinase K or porcine pepsin, in the agar well diffusion assay abrogated the inhibitory effect showing the antifungal activity was no longer evident (Figure 1). This indicated that the phenolic compound contaminants in the precipitated protein did not contribute to the observed anti-fungal activity and that the anti-fungal activity was either solely due to or dependent on protein(s) in the extract.

### 3.2. Antifungal Activity of The Protein Fractions Against T. marneffei

To begin the process of identifying the active agent(s) in the *R*. *nasutus* protein extract, the proteins were separated into 3 fractions by membrane cut-off centrifugation. Each fraction contained proteins with molecular weight ≥30 kDa (RN_A), 10–30 kDa (RN_B), and ≤10 kDa (RN_C). Initially, anti-*T. marneffei* activity was tested in the microbroth dilution methods against both yeast and mycelia of *T. marneffei* using 200 µg/mL of the protein fractions. However, only the yeast phase was sensitive in the assay, so the yeast phase was used for all subsequent antifungal activity testing. Sensitivity testing was then conducted by the microbroth dilution assay using a series of fraction concentrations from 0.25–128 µg/mL. All fractions possessed the anti-*T. marneffei* activity, exhibiting MIC and MFC values at 32–128 μg/mL and >128 μg/mL, respectively (Table 3).

### 3.3. Effects of R. nasutus Anti-Fungal Activity in T. marneffei Mutant Strains

#### 3.3.1. Mutants in Gα Subunits of Heterotrimeric G-Proteins

**Figure 2:**
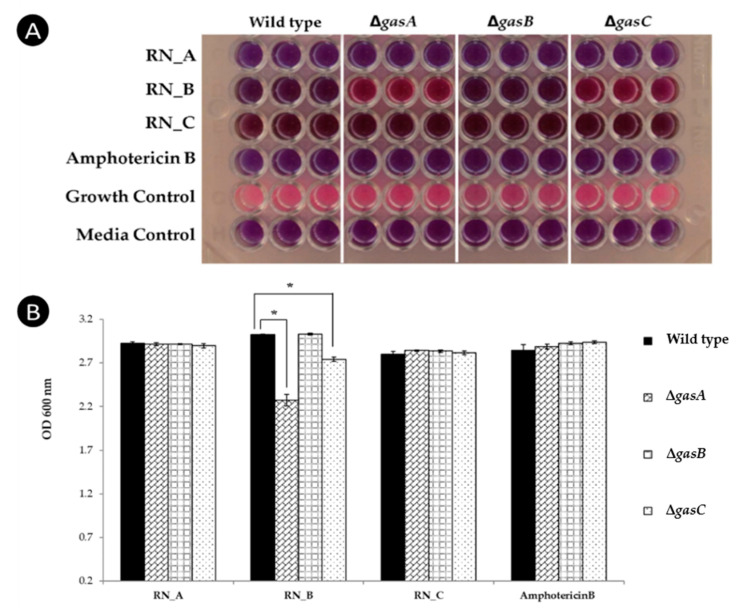
Effect of mutations affecting G-protein signaling in *T. marneffei* on anti-fungal susceptibility. *T. marneffei* yeast cells (10^4^ cells/mL) of the wild type, Δ*gasA*, Δ*gasB* and Δ*gasC* strains were treated with 4xMIC of the RN_A, RN_B and RN_C fractions and incubated for 72 h at 37 °C. (**A**) Broth microdilution assay showed the growth of Δ*gasA* and Δ*gasC* mutants (pink) in treatment with RN_B fraction but no growth (purple) for the Δ*gasC* and wild type strains. (**B**) The level of growth in each well was quantified by OD_600_ measurement. Asterisks (*) shows significantly difference (*p* < 0.05).

*Penicillium chrysogenum* antifungal peptide (PAF), a well-characterized antifungal peptide from *Penicillium chrysogenum* activates the cAMP/PKA signaling cascade via heterotrimeric G-protein at the cell membrane when tested in *Aspergillus nidulans*, which leads to apoptosis-like phenomenon [37,38]. To determine whether the anti-*T*. *marneffei* activity of *R. nasutus* proteins also act through G-protein signaling, they were tested for antifungal activity against a panel of mutant strains by broth dilution assay. The yeast cells of *T. marneffei* strains carrying mutations in each of the three genes that encode alpha subunits of heterotrimeric G proteins; Δ*gasA*, Δ*gasB,* and Δ*gasC*, were subjected to treatment with each of the protein fractions at 4xMIC. The growth percentage of the mutant strains was compared to that of the wild type. From the results, there was no significant difference in growth when comparing the wild type to the Δ*gasA*, Δ*gasB,* and Δ*gasC* mutants in the absence of any inhibitory compounds, as shown by the pink color indicating fungal growth (Figure 2). All mutants showed susceptibility to the amphotericin B positive control (purple color indicating cell death). For treatment with *R. nasutus* protein fractions, the wild type and Δ*gasB* mutant showed susceptibility to all protein fractions while the Δ*gasA* and Δ*gasC* mutants grew in the treatment with RN-B (Figure 2A). The fungal growth in each well was quantified by OD_600_ measurement (Figure 2B). Significant growth reduction was observed when Δ*gasA* and Δ*gasC* were treated with protein fractions. These results indicated that GasA and GasC alpha subunits are important to the antifungal activity of the proteins in the RN-B. The active proteins in this fraction should be further identified.

**Figure 3:**
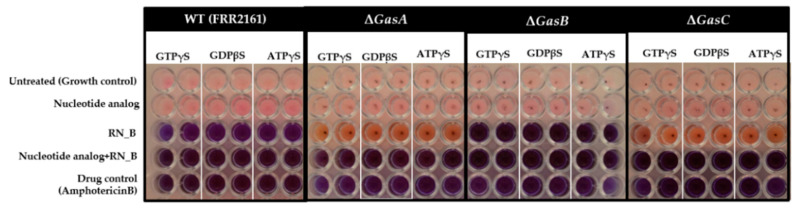
Effect of the guanidine nucleotide analogues on G-protein signaling in the presence of the anti-*T. marneffei* RN_B fraction. Yeast cell (10^4^ cells/mL) of *T. marneffei* wild type, Δ*gasA*, Δ*gasB* and Δ*gasC* strains were treated with nucleotide analogues (guanidine nucleotide analogues, guanosine-5′-(3-thiotriphosphate) (GTPγS), guanosine-5′-*O*-(2-thio-bisphosphate) (GDPβS) and adenosine 5′-[γ-thio]triphosphate (ATPγS)) at 37 °C, 15 min before added the RN_B fraction and incubated for 72 h at 37 °C. Pink and purple indicated growth and no growth of *T. marneffei*, respectively.

To confirm that the activity of the RN_B fraction affected the G-protein signaling pathway, guanidine nucleotide analogues were tested in the antifungal assay. Treatment of the *T. marneffei* strains with the guanidine nucleotide analogues GTPγS and GDPβS. GTPγS is a non- or slowly hydrolysable form of GTP that maintains G-protein signalling in the active state while GDPβS blocks G-protein signalling. The ATPγS nucleotide analogue, which does not perturb G-protein signalling, was used as a control. In the presence of the analogues only, there was no effect on *T. marneffei* growth for either the wild type or any of the mutant strains as measured in this assay (Figure 3, pink). In the presence of the RN_B fraction, the wild type strain could not grow (purple), whereas only partial inhibition (orange) was observed for the Δ*gasA* and Δ*gasC* strains but not Δ*gasB* (Figure 3). The basis for the partial restoration of growth, as opposed to complete restoration, may be due to the fact that RN-B contains a number of different proteins, some of which may also have anti-fungal activity but not operate through G-protein signaling or that *gasA* and *gasC* are partially redundant. When both the nucleotide analogues and RN_B fraction were used, the wild type and Δ*gasB* strains remained inhibited, while the effects of the Δ*gasA* and Δ*gasC* mutations on RN_B-associated growth inhibition was lost, and the strains displayed strong growth inhibition. This suggests that the antifungal proteins in the RN_B fraction act through the GasA and GasC alpha subunits or are dependent on the associated G-protein signaling pathway. It is important to note that ATPγS showed similar effects to both the guanine nucleotide analogues, suggesting that the mechanism of action of these analogues may not be due to the expected targeting of G-alpha activity.

**Figure 4:**
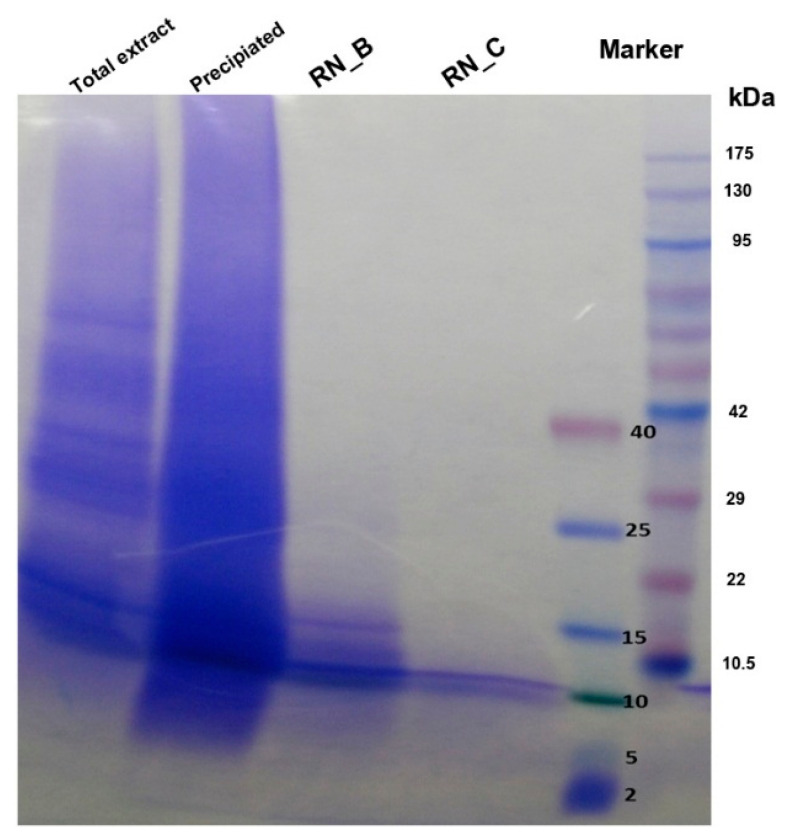
SDS-PAGE analysis of the *R. nasutus* proteins. Five micrograms of each protein extract were fractionated on a gradient 4–20% polyacrylamide gel. Lane 1: Total protein extract, Lane 2: Ammonium precipitated protein extract, Lane 3: RN_B fraction, Lane 4: RN_C fraction, Lane 5: Spectra^TM^ multicolor low range protein ladder (Thermo Scientific, Rockford, IL, USA), Lane 6: Pink plus prestained protein ladder (GeneDirectX Inc., Warwickshire, UK). Protein bands were detected by Coomassie blue-R250 staining.

The protein extracts were examined by SDS-PAGE analysis, which showed that RN_B fraction contained a number of distinct peptides (Figure 4). The RN_C fraction also showed a number of distinct peptides, but fewer than RN_B, that overlapped in size with those noted in RN_B. It remains unresolved whether one or more than one of the observed peptides is responsible for the anti-fungal activity.

#### 3.3.2. Mutants in The Cell Wall Integrity Pathway

**Figure 5:**
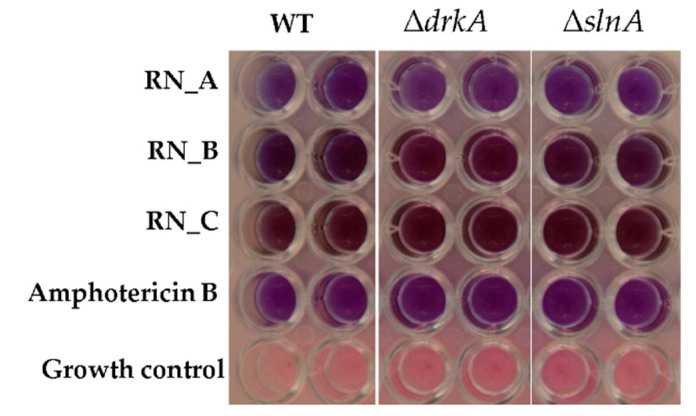
Effect of mutation of the *slnA* and *drkA* mediated cell wall integrity pathway on the anti-*T. marneffei* activity. Yeast cells (10^4^ cells/mL) of the wild type, Δ*drkA* and Δ*slnA T. marneffei* strains were treated with RN_A, RN_B and RN_C fractions at 4x of the MIC and incubated for 72 h at 37 °C. Pink and purple colours indicated growth and no growth of *T. marneffei*, respectively.

In *T. marneffei*, the SlnA and DrkA hybrid histidine kinases are the first components of a phosphorelay system that transfers signals from cell membrane to nucleus via a mitogen-activated protein kinase (MAPK). This signaling pathway is important in cell wall integrity by sensing environmental changes and controlling the expression of genes that function in cell wall rearrangement [36]. The Δ*slnA* and Δ*drkA* mutant strains were used to investigate whether the antifungal proteins in RN_B fraction could target the cell-wall integrity pathway either directly via the hybrid histidine kinase sensors or indirectly. The wild type, Δ*slnA* and Δ*drkA* strains were assayed for growth after treatment with protein fractions from *R. nasutus* (Figure 5). The three fractions of the *R. nasutus* extract were tested for the antifungal activity using the 10^4^ yeast cells. After incubation at 37 °C for 72 h, the results showed no difference between the wild type control and either of the Δ*slnA* and Δ*drkA* strains. Therefore, the anti-*T*. *marneffei* activity was not dependent on the SlnA and DrkA histidine kinase pathway.

## 4. Discussion

*Rhinacanthus nasutus* is a well-known, versatile plant that has been used for treatment of various infectious diseases and cancers in Thai alternative medicine for many years [17]. This plant has also been reported to contain antifungal activity [18]. Rhinacanthins are known antimicrobial compounds from *Rhinacanthus* and, although they demonstrate highly effective antimicrobial properties, they can also be highly toxic due to their chemical nature [17]. In this work, we demonstrated for the first time that *R. nasutus* contained antifungal proteins that inhibited the growth of the opportunistic dimorphic fungus *T. marneffei*. In this study, total proteins were separated into three fractions according to their size into ≥30 (RN_A), 10-30 (RN_B), and ≤10 kDa (RN_C), and SDS-PAGE analysis showed some overlap in proteins amongst the fractions as gauged by size. The broth microdilution assay revealed that all fractions possessed anti-*T. marneffei* activity as assessed by growth inhibition. Although the observed activity was not yet comparable to the potency of the conventional drug amphotericin B it provides a starting point for the development of new antifungal compounds. The data showed that there were different MICs among three fractions. This result suggests either that different types of antifungal peptides were presented in each fraction or the same peptide or derivative was present in different concentrations. Further purification of the active peptide(s) from the extracts is crucial to understand whether there is one or more active AFPs in the preparation and to begin to address the possible modes of action.

A possible mechanism underlying the anti-*T. marneffei* activity was investigated in this study. Adopting knowledge from a well-characterized PAF antifungal peptide from *Penicillium chrysogenum*, which activates the heterotrimeric G-proteins and leads to apoptotic-like death of the fungal cells [37,38], we questioned whether the RN_B fraction might act in a similar manner via the G-protein signaling pathway. *T. marneffei* has three Gα subunit encoding genes (*gasA*, *gasB*, and *gasC*), one Gβ subunit-encoding gene (*sfaD*), and one Gγ subunit-encoding gene (*gpgA*) [33,34,35]. The genes encoding Gα subunits are of particular interest since these subunits govern their specific biological function. Specifically, GasA is a key regulator of conidiation, GasB is involved in the regulation of growth in response to pH, and GasC affects conidial germination and is involved in the regulation of secondary metabolite production [33,34,35]. Antifungal activity testing of the *R. nasutus* extracts against Δ*gasA*, Δ*gasB*, and Δ*gasC* mutant strains demonstrated that the AFPs of size range 10–30 kDa (RN_B) showed differential effects on the GasA and GasC Gα subunit mutants but not on GasB.

We also examined the RN_B activity on trimeric G-protein signalling by using nucleotide analogues in the antifungal testing regimen. Normally, the activation of G-protein receptors by agonists leads to the dissociation of GDP from Gα subunit, followed by the binding of GTP, leading to an activated state. Subsequent hydrolysis of GTP assists in modulation of the downstream effectors. In nucleotide analogues assay, the GPCR activity is affected by these stable guanidine nucleotide analogues GTPγS and GDPβS [39]. Our results showed that the addition of guanine nucleotide analogues to the wild type strain did not recapitulate the reduced sensitivity to the RN_B fraction noted for the Δ*gasA* and Δ*gasC* mutants, suggesting that either the nucleotide analogues cannot access their target or inhibition of Gα activity by these analogues is not equivalent to complete loss of the Gα proteins with respect to sensitivity to the RN_B fraction. The observation that the anti-*T. marneffei* activity of RN_B to Δ*gasA* and Δ*gasC* mutants is partially restored in the presence of nucleotide analogues may suggest access of the nucleotide analogues to their target is not an issue and that the RN_B peptides act through the GasA and GasC or, based on the adenosine analogue also showing this effect, that the mechanism is unrelated to direct effects of the state of the G alpha. In contrast, when mutants affecting the cell wall integrity pathway, Δ*slnA* and Δ*drkA*, were assessed in the antifungal assay, there was no observable difference in inhibition by the RN_B peptides compared to the wild type, indicating the anti-*T*. *marneffei* activity was not dependent on the SlnA and DrkA mediated histidine kinase signalling pathway. It is important to note that there are seven genes encoding hybrid histidine kinases in *T. marneffei* and any redundancy has not been examined. Further investigations with more mutants in this cell wall integrity pathway could provide the better answers.

## 5. Conclusions

In summary, this study reports on promising antifungal proteins present in the medicinal plant *R. nasutus*. These AFPs may be candidates for drug development in order to address to unmet need for new anti-fungal agents to treat fungal infections. The observation that these AFPs may act through the well-studied G proteins signaling pathway, which is important for several biological functions including pathogenesis, and that they are diverged from the mammalian counterparts makes them important candidates [33,34,35,40].

## Figures and Tables

**Table 1:** Fungal strains used in this study.

Strain	Product	Role(s)	Reference
FRR2161	Wild type	American Type Culture Collection strain (ATCC18224)	
Δ*gasA*	Gα subunit	Negative regulation of asexual development, positive regulation of secondary metabolism	Zuber et al., 2002 [33]
Δ*gasB*	Gα subunit	The regulation of growth in response to pH	Zuber, 2003 [34]
Δ*gasC*	Gα subunit	The major regulator of conidial germination	Zuber et al., 2003 [35]
Δ*drkA*	Hybrid histidine kinases (HHK) (class III)	Hyphal morphogenesis; response to osmotic, oxidative, and cell wall stress; resistance to antifungal agents; promotion of asexual development; production of yeast cells (dimorphic switching)	Boyce et al., 2011 [36]
Δ*slnA*	Hybrid histidine kinases (HHK) (class VI)	Germination; hyphal morphogenesis; response to osmotic, oxidative and cell wall stress; promotion of asexual development	Boyce et al., 2011 [36]

**Table 2:** Antifungal activity of *R. nasutus* precipitated proteins against *T. marneffei.*

Substance	Antifungal Activity (Mean ± SD of Inhibition Zone Diameter (mm))
*Rhinacanthus nasutus* protein extract	+ (16.00 ± 0.00)
Negative control (10 mM CH_3_COONa)	−
Positive control (10 µg/mL amphotericin B)	+ (18.75 ± 0.43)

+: inhibition, −: no inhibition. Value are means ± SD (Standard deviation) (n = 3).

**Table 3:** The minimal inhibitory concentration (MIC) and minimal fungicidal concentration (MFC) values of the protein fractions against *T. marneffei*.

Fraction	MIC (μg/mL)	MFC (μg/mL)
RN_A	32	>128
RN_B	64	>128
RN_C	128	>128
Amphotericin B (10 µg/mL)	0.3125	0.5

RN: *Rhinacanthus nasutus*, RN_A; ≥30 kDa, RN_B; 10 to 30 kDa, RN_C; ≤10 kDa.
